# Characterization of a Thermo-Inducible Chlorophyll-Deficient Mutant in Barley

**DOI:** 10.3389/fpls.2017.01936

**Published:** 2017-11-14

**Authors:** Rong Wang, Fei Yang, Xiao-Qi Zhang, Dianxin Wu, Cong Tan, Sharon Westcott, Sue Broughton, Chengdao Li, Wenying Zhang, Yanhao Xu

**Affiliations:** ^1^Hubei Collaborative Innovation Centre for Grain Industry and Hubei Key Laboratory of Waterlogging Disaster and Agriculture Use of Wetland, College of Agriculture, Yangtze University, Jingzhou, China; ^2^Western Barley Genetics Alliance, Murdoch University, Murdoch, WA, Australia; ^3^Department of Genetics and Cell Biology, Yangtze University, Jingzhou, China; ^4^Western Australian Agricultural Biotechnology Centre, Murdoch University, Murdoch, WA, Australia; ^5^Institute of Nuclear Agricultural Science, Zhejiang University, Hangzhou, China; ^6^Agriculture and Food, Department of Primary Industries and Regional Development, South Perth, WA, Australia

**Keywords:** barley, thermo-inducible chlorophyll-deficient mutant, fine mapping, QTL, *vvy* gene

## Abstract

Leaf color is an important trait for not only controlling crop yield but also monitoring plant status under temperature stress. In this study, a thermo-inducible chlorophyll-deficient mutant, named V-V-Y, was identified from a gamma-radiated population of the barley variety Vlamingh. The leaves of the mutant were green under normal growing temperature but turned yellowish under high temperature in the glasshouse experiment. The ratio of chlorophyll *a* and chlorophyll *b* in the mutant declined much faster in the first 7–9 days under heat treatment. The leaves of V-V-Y turned yellowish but took longer to senesce under heat stress in the field experiment. Genetic analysis indicated that a single nuclear gene controlled the mutant trait. The mutant gene (*vvy*) was mapped to the long arm of chromosome 4H between SNP markers 1_0269 and 1_1531 with a genetic distance of 2.2 cM and a physical interval of 9.85 Mb. A QTL for grain yield was mapped to the same interval and explained 10.4% of the yield variation with a LOD score of 4. This QTL is coincident with the *vvy* gene interval that is responsible for the thermo-inducible chlorophyll-deficient trait. Fine mapping, based on the barley reference genome sequence, further narrowed the *vvy* gene to a physical interval of 0.428 Mb with 11 annotated genes. This is the first report of fine mapping a thermo-inducible chlorophyll-deficient gene in barley.

## Introduction

Chlorophyll plays a unique and indispensable role for energy transfer in the reaction centers of photosynthesis (Tanaka and Tanaka, 2011; Lin et al., 2014). There are two types of chlorophyll in plants, chlorophyll *a* and chlorophyll *b*. Chlorophyll *a* is the principal photosynthetic pigment, harvesting energy directly from the sun, while chlorophyll *b* is an accessory photosynthetic pigment, transferring light energy to chlorophyll *a*. Chlorophyll content is a crucial trait for crop biomass and grain yield. Chlorophyll biosynthesis and biodegradation pathways are significant biochemical pathways and have received much attention from biochemists (Tanaka and Tanaka, 2007; Hörtensteiner and Kräutler, 2011).

Many types of chlorophyll-deficient mutants have been reported in plants and subdivided into four types based on the physiological mechanisms involved: total chlorophyll increase type, total chlorophyll deficiency type, chlorophyll *a* deficiency type and chlorophyll *b* deficiency type (Falbel and Staehelin, 1996). Thermo-sensitive chlorophyll mutants are a particular type of virescence, exhibiting normal or near-normal phenotypes at permissive temperatures, but mutational phenotypes at lower or higher temperatures.

Studies have shown that chlorophyll content is associated with plant heat stress (Havaux and Tardy, 1999; Mohammadi et al., 2009; Jespersen et al., 2016; Sun and Guo, 2016; Rossi et al., 2017), and chlorophyll fluorescence is a key indicator for monitoring the physiological status of plants under temperature stress (Jespersen et al., 2016; Oukarroum et al., 2016) and for screening heat-tolerant cultivars (Ristic et al., 2007; Kalaji et al., 2011; Sharma et al., 2012; Chen et al., 2016). Thus, thermo-sensitive mutants are of particular interest for studying chlorophyll biogenesis in response to temperature cues.

Mutants are key resources in gene cloning, functional analysis, and the elucidation of biochemical processes and pathways. In rice, more than 170 leaf color mutations have been described, and more than 160 leaf color genes have been identified across all 12 chromosomes, of which about 40 genes are directly involved in chlorophyll biosynthesis and/or catabolism (Deng et al., 2014; Zhu et al., 2016). In barley, a large number of mutant phenotypes with full or partial chlorophyll deficiency have been described (Gálová et al., 2000; Franckowiak and Lundqvist, 2012) but only a small number of genes were identified. Fructokinase-1-like (FLN) was identified as a candidate gene for barley stage green-revertible albino (*HvSGRA*) (Qin et al., 2015). A 95 bp insertion in proto chlorophyllide oxidoreductase (POR) resulted in chlorophyll deficiency in the NYB mutant barley (Liu Z.L. et al., 2008). Recently, genes controlling chlorophyll synthesis have been explored by RNA-seq in barley *Alm* gene near-isogenic lines (Shmakov et al., 2016). However, the genes responsible for chlorophyll biosynthesis and biodegradation and leaf color formation are largely unknown in barley.

Thermo-sensitive chlorophyll mutants have been reported in *Arabidopsis thaliana* (Markwell and Osterman, 1992), rice (Dong et al., 2001), maize (Pasini et al., 2005) and barley (Gálová et al., 2000). In rice, five genes participated in the temperature-induced abnormal leaf phenotype regulation have been cloned, including genes *OsGluRS* (Liu et al., 2007), *tscd1* (Liu et al., 2015), *tcd5* (LOC_Os05g34040) (Wang Y. et al., 2016) and *NOA1* (Yang et al., 2011). In *Arabidopsis thaliana*, the genes CHLOROPHYLL SYNTHASE (*chlg1-1*) (Lin et al., 2014) and CHLOROPHYLL DEPHYTYLASE1 (*cld1-1*) (Lin et al., 2016) responsible for cotyledon-bleaching phenotype have been cloned. Barley chlorophyll mutants have displayed various temperature stress response patterns (Gálová et al., 2000). However, the genes and mechanisms of sense and response to temperature in barley chlorophyll biosynthesis are largely unknown.

Barley, as one of the most important sources for brewing and feeding in the world, is also a model plant for researchers in Triticale species with large genomes. The recent release of the barley genome sequence will accelerate study and cloning of genes relevant to agriculture (Mascher et al., 2017). In this study, a thermo-inducible chlorophyll-deficient mutant (V-V-Y) was identified from the cultivated barley Vlamingh using gamma-radiation treatment. The mutant displayed normal leaf color at permissive temperatures but turned yellowish when the temperature was above 30°C. We conducted genetic analysis of the chlorophyll-deficient mutant gene (*vvy*) and constructed a genetic map of the *vvy* gene. New markers were developed to fine mapping the *vvy* gene based on the barley reference genome sequence and whole-genome shotgun sequences of the two parents. Additionally, quantitative trait loci (QTL) analysis was performed to understand the effect of the mutant gene on yield.

## Materials and Methods

### Plant Materials

The mutant V-V-Y, which exhibits the thermo-inducible chlorophyll-deficient phenotype, was identified from a 200 gray gamma-radiation treatment of barley cultivar Vlamingh, a malting barley bred by the Department of Agriculture and Food Western Australia. The leaves of the mutant V-V-Y are green at normal temperature, but yellowish at temperatures above 30°C. A double haploid (DH) population with 203 lines was developed by anther culture from the F_1_ lines of V-V-Y × Buloke. Buloke is an Australian two-rowed malting barley with similar maturity, good agronomic traits, and green leaves.

### Glasshouse Experiments

The 203 DH lines, mutant V-V-Y, and barley varieties Buloke and Vlamingh were planted in pots in a glasshouse at the Western Australian State Agricultural Biotechnology Centre. A high-temperature treatment of 30°C for 18 days was applied at seeding and tillering stages. After thermal treatment, the leaf color of each line was observed every 2 days. The DH lines with consistent phenotypes at both stages were used for mapping analysis.

### Chlorophyll Content Measurements

To confirm the effect of temperature on chlorophyll content in the mutant V-V-Y, the chlorophyll *a* and chlorophyll *b* contents were determined with a spectrophotometer according to the method of Hiscox and Israelstam (1979). Approximately 0.5 g fresh leaves were cut into pieces and homogenized in 7 ml dimethyl sulfoxide (DMSO) and incubated at 65°C for 1 h. The extract was used to measure the absorbance value at 645 and 663 nm with a pure DMSO solution as a control. Dilute 5–10 times, if necessary. Each leaf sample was assayed with three replicates. Chlorophyll *a* and *b* contents were calculated as follows:

Chlorophyll *a* (g/L) = 0.0127A663–0.00269A645
Chlorophyll *b* (g/L) = 0.0029A663–0.00468A645

The unit of chlorophyll: mg/g fresh weight = (chlorophyll *a* or chlorophyll *b* × volume (L) × dilute times)/sample weight (g).

### Genetic Linkage Map Construction

Genomic DNA was extracted using the SDS method (Edwards et al., 1991). The Fluidigm SNP genotyping system (Fluidigm, South San Francisco, CA, United States) was used to screen 446 previously designed SNP markers for polymorphism between parents (Close et al., 2009). The selected polymorphic markers between V-V-Y and Buloke were used to acquire genotype data of the DH population via the Fluidigm SNP genotyping system. The genotypic data was collected and inputted into JoinMap 4.1 software for marker linkage group analyses. The multipoint maximum likelihood mapping algorithm was used to calculate genetic distances with a LOD score > 3. The SNP marker linkage map was created using the integrated MapChart program in JoinMap (Voorrips, 2002). The final map was validated according to the barley reference genome sequence (Mascher et al., 2017).

### Fine Mapping of *vvy* Gene and Candidate Gene Analysis

InDel markers were designed in the preliminary mapped region based on sequence alignments of Scope and Vlamingh with the Morex reference genome sequence. Scope was derived from Buloke with a single SNP variation for herbicide tolerance and thus used as the reference sequence for Buloke. The whole-genome shotgun sequences of Vlamingh and Scope were generated in-house and are available by request. The polymorphic InDel markers were used to analyze the genotype of the DH population (**Table 1**). Genotyping was performed by standard PCR. The PCR products were run on either 2.5% agarose gel or 6% sodium dodecyl sulfate-polyacrylamide gel. The phenotypic data and the genotypic date were combined for mapping of *vvy* gene according to the above method.

**Table 1:** The polymorphic InDel markers screened from the preliminary mapped region of the *vvy* gene used for fine mapping.

Name	Sequence (forward/reverse 5′ to 3′)	Chr 4H position (Mb)
Indel4157	GCCATCGTTCGGCCATCTTT/TGGATTCCCTTTGACTGCACA	635.50
WR13	CTGAGAAGCATATCGTGAATGG/ACAGGAAGACGATCTCGTTG	636.21
WR15	GATGATGATCGTGGATGGAACT/TAGGCACAACGCTTTTACTGGT	636.21
WR17	TCGTGGTCGACACTGAGATTAC/TCGTGGTCGACACTGAGATTAC	638.07
WR21	GAAGGTAAACGCTGATAGGGTG/CCCAATCTTCCATGTAGGAGGT	638.11
WR68	GAAAAATGGTGGGGTTTATTCA/CGACAAATTATTGCTGGAAAA	640.65
WR12	ACATACGGTGGGTCGACAAA/CCAAGGAAAAATGGTGGGGT	640.66
WR76	ATGAAACACCGTTGCTCCTTAT/AGAGGTGTTTTGGCTCCAGAT	641.08
WR25	ACCTGTCTGTCTGTCTGTCACG/AAGGAATCCGCAGATCACATAC	641.09
WR26	TGCATGCGATCAGATGATTTAT/ACAAGTCCAGAGGACACACGTA	641.90
WR33	CCCCACAATTCTCTGTTGATTT/GCATGACGACGATCACTCTGT	642.50
WR37	TGTAAAGTGTCTTCTTCGATGGG/GGTGGTTTGTTTTCTGAGGACT	645.60

Information on the candidate genes in the target genetic region was extracted from the new barley genome annotation (Mascher et al., 2017). The amino acid sequence encoded by each candidate gene was searched for homologs using the UniProt database^1^. Moreover, the reviewed entry hits from UniProt were used as references for the biological functional prediction. Functional domain analyses were performed using the InterPro online tool^2^.

### QTL Analysis for Yield

Vlamingh and its mutant V-V-Y were planted in 1 m long double rows with row spacing of 30 cm at Zhejiang University field stations. The materials were exposed to temperatures over 36°C for 2 days, at the later dough stage, under natural conditions. The yield trial was conducted under typical Mediterranean climate conditions with terminal drought and heat stresses to favor the trait expression. The DH population and parents were planted in 1 × 5 m plots in a randomized complete block design. Only the middle six rows (1 × 3 m) were harvested to evaluate yield. Parental and local barley varieties were used as grid controls for spatial analysis. The yield was adjusted according to the Best Linear Unbiased Prediction (BLUP) (Liu X.Q. et al., 2008). Individual yield data in the V-V-Y × Buloke double haploid population were used for QTL analysis. The software package MapQTL 5.0 (Van Ooijen, 2004) was used to conduct QTL analysis for barley yield after importing the files for genotypes, phenotypes, and genetic maps. Composite interval mapping (CIM) was used for QTL mapping. LOD threshold values declaring the presence of a QTL were estimated by performing whole-genome-wide permutation tests using 1,000 permutations. The QTL map was then generated using MapChart 2.2.

## Results

### Phenotypic Characterization of the Mutant V-V-Y

**FIGURE 1:**
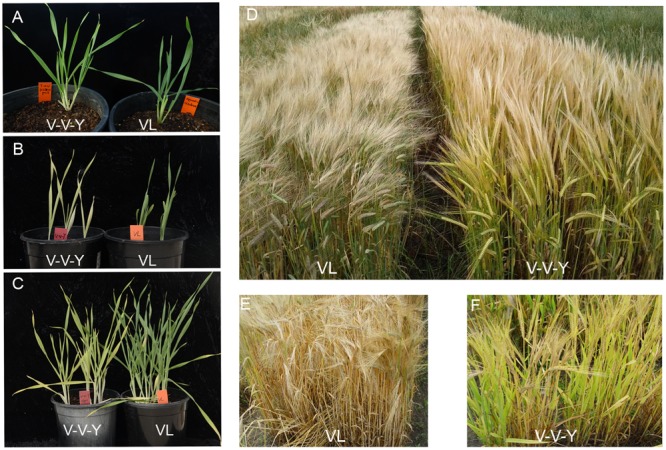
Leaf colors in the mutant V-V-Y and the wild parent Vlamingh (VL) in the glasshouse and field. **(A)** leaf colors of VL and V-V-Y at the seeding stage at normal temperature, **(B)** leaf colors of VL and V-V-Y after 12 days of high-temperature treatment (30°C) at the seeding stage in the glasshouse, **(C)** leaf colors of VL and V-V-Y after 12 days of high-temperature treatment (30°C) at the tillering stage in the glasshouse, **(D)** plot performance of VL and V-V-Y at the grain-filling stage in the field under high temperature, **(E)** leaf color of VL at the grain-filling stage in the field under high temperature, **(F)** leaf color of V-V-Y at the grain-filling stage in the field under high temperature.

The leaves of both V-V-Y and Vlamingh are green at normal temperature (**Figure 1A**). In the glasshouse at high temperature (30°C), the leaves of V-V-Y started turning yellowish after 5 days and were fully yellow after 12 days treatment at both the seeding stage (**Figure 1B**) and the tillering stage (**Figure 1C**). In the field, when high temperatures occurred during the grain-filling stage, the leaves of V-V-Y turned yellowish (**Figures 1D,F**), while those of the parent Vlamingh turned yellow (**Figures 1D,E**). The leaves of V-V-Y took longer to senesce than the parent Vlamingh during the grain-filling stage at high temperature (**Figures 1E,F**).

### Chlorophyll Content in V-V-Y under High Temperature

**FIGURE 2:**
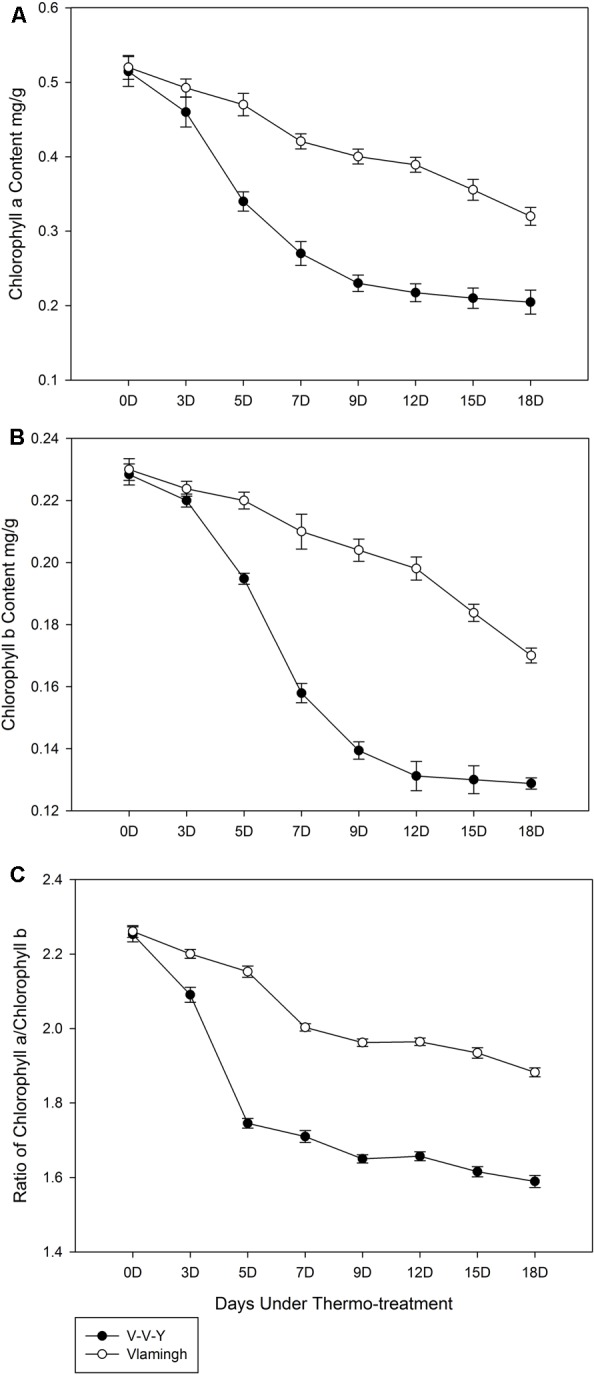
Chlorophyll contents of V-V-Y and Vlamingh at the tillering stage in the glasshouse high-temperature treatment (30°C). **(A)** chlorophyll *a* contents in V-V-Y and Vlamingh after 3, 5, 7, 9, 12, 15, and 18 days of high temperature, **(B)** chlorophyll *b* contents in V-V-Y and Vlamingh after 3, 5, 7, 9, 12, 15, and 18 days of high temperature, **(C)** the ratio of chlorophyll *a*/chlorophyll *b* in V-V-Y and Vlamingh after 3, 5, 7, 9, 12, 15, and 18 days of high temperature.

Under the high-temperature treatment, chlorophyll *a* content in Vlamingh declined by 0.011 mg g^-1^ leaf per day (**Figure 2A**), which showed a good linear relationship with the treatment time (*y* = -0.0279x + 0.5468, *R*^2^ = 0.9869, “y” is the chlorophyll *a* content in Vlamingh, “x” is the treatment day). In V-V-Y, the chlorophyll *a* content declined by 0.017 mg g^-1^ leaf per day, on average; notably, the rate of decline in the first 7 days was five times higher than the last 11 days (**Figure 2B**). Under high-temperature treatment, the average rate of decline in chlorophyll *b* content in V-V-Y was about 1.6 times higher than in Vlamingh (**Figure 2B**) and markedly higher in the first 9 days (**Figure 2B**).

The ratio of chlorophyll *a*/chlorophyll *b* in Vlamingh remained relatively stable with a slight reduction throughout the high-temperature treatment, while that of V-V-Y declined more sharply in the first 5 days of treatment and remained relatively stable with a slight reduction over the next 13 days (**Figure 2C**). V-V-Y had a lower chlorophyll *a*/chlorophyll *b* ratio than Vlamingh throughout the high-temperature treatment, indicating that chlorophyll *a* declined faster than chlorophyll *b* in V-V-Y.

### Inheritance and Preliminary Mapping of *vvy* Gene

In the V-V-Y × Buloke DH population, the high-temperature treatments in the glasshouse identified 100 individuals with the mutant phenotype of thermo-inducible yellowish leaf color and 103 individuals with the wild phenotype of green leaf color under high-temperature stress. Chi-square analysis showed that the ratio of mutant phenotype type: wild phenotype type fit the 1: 1 segregation ratio (*χ*^2^ = 0.0225, *P* > 0.90), revealing that the thermo-inducible yellowish leaf color phenotype is governed by a single mutant gene in V-V-Y.

**FIGURE 3:**
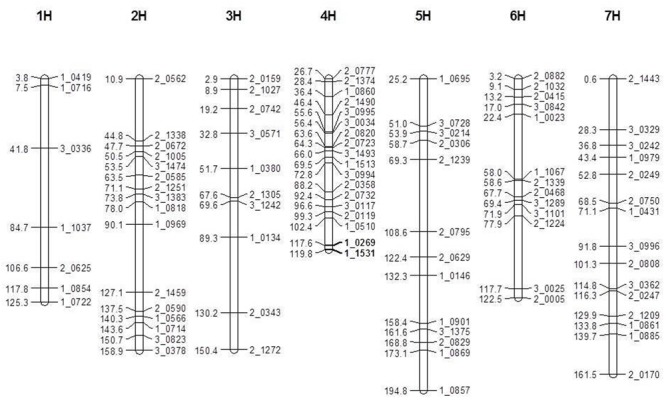
Linkage map of the V-V-Y × Buloke DH population on the barley 7 chromosomes and preliminary mapping interval of *vvy* gene. Marker locus names are to the right of each linkage group and distances (Kosambi cM) are to the left of each marker interval. Solid black bars indicate the preliminary mapping interval of *vvy* gene.

A total of 446 pairs SNP markers were tested for polymorphism between V-V-Y and Buloke. Ninety-six polymorphic SNP markers were selected for genetic map construction. The total length of the genetic linkage map was 1033 cM with an average distance of 11.3 cM between two markers (**Figure 3**). The mutant gene (*vvy*) controlling the thermo-inducible yellowish leaf color phenotype in V-V-Y was mapped to the terminal region of the long arm of chromosome 4H between the SNP markers 1_0269 and 1_1531 with a genetic distance of 2.2 cM. These two molecular markers were located at 636.35 and 646.20 Mb of chromosome 4H in the new barley reference genome sequence (Mascher et al., 2017). The preliminary physical region of the *vvy* gene was mapped to a 9.85 Mb region.

### Fine Mapping of *vvy* Gene and Candidate Gene Analysis

**FIGURE 4:**
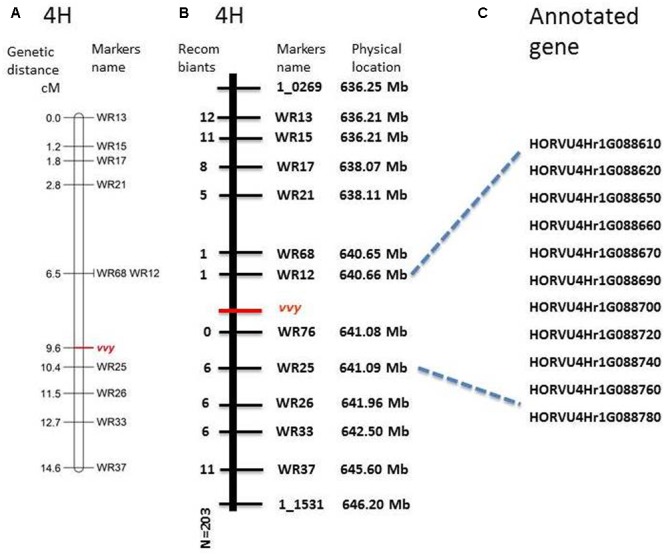
Fine mapping of *vvy* gene on barley chromosome 4H and the candidate genes. **(A)** Linkage map of *vvy* gene, marker locus names are to the right of each linkage group and distances (Kosambi cM) are to the left of each marker interval, **(B)** physical mapping, marker names and the physical location on the right, the number of detected recombinant individuals on the left, **(C)** predicated candidate genes.

To fine map the *vvy* gene, 117 InDel markers were designed within the preliminary mapped region. Polymorphism was screened between the parent lines, which yielded 12 polymorphic markers (**Table 1**). These markers were used to genotype the V-V-Y × Buloke DH population. The linkage and fine mapping results showed that the *vvy* gene was located between marker WR12 (640.660333 Mb) and marker WR25 (641.088341 Mb), with a physical interval of 0.428 Mb (**Figures 4A,B**).

According to the most recent barley genome annotation (Mascher et al., 2017), 11 candidate genes are located in this region (**Figure 4C** and **Table 2**). The predicted protein function and biological process for each candidate gene are listed in **Table 2**. Of these 11 genes, HORVU4Hr1G88650 is the kinase family protein, HORVU4Hr1G88690 is the transcription factor, HORVU4Hr1G88670 has unknown function, HORVU4Hr1G88700 controls lateral root development, and the other seven participate in several biochemical metabolism processes (**Table 2**). It should be noted that HORVU4Hr1G88610 was predicted to target the chloroplast^3^.

**Table 2:** List of candidate genes in the target genetic region.

No.	Gene ID	4H location (bp)	Protein	Biological process
1	HORVU4Hr1G088610	640662818-640665994	Quinone oxidoreductase	ATP synthesis coupled electron transport
2	HORVU4Hr1G088620	640666350-640668741	Phosphoglycerate mutase family protein	Probable helicase-like transcription factor
3	HORVU4Hr1G088650	640685328-640696684	Protein kinase family protein	Sugar transport symport transport
4	HORVU4Hr1G088660	640701625-640703561	E3 ubiquitin-protein ligase PRT6	Lipid catabolic process, seed germination, ubiquitin-dependent protein catabolic process
5	HORVU4Hr1G088670	640704613-640707799	Unknown function	Unknown function
6	HORVU4Hr1G088690	640711150-640712652	General transcription factor IIH subunit 5	Transcription and DNA repair factor
7	HORVU4Hr1G088700	640716952-640729169	ARABIDILLO-1	Lateral root development
8	HORVU4Hr1G088720	640726929-640730325	Palmitoyl-protein thioesterase 1	Palmitoyl hydrolase activity
9	HORVU4Hr1G088740	640731612-640733092	Class I glutamine amidotransferase-like superfamily protein	Leaf senescence
10	HORVU4Hr1G088760	641042190-641047868	Long chain base biosynthesis protein 2d	Liquid metalism
11	HORVU4Hr1G088780	641086962-641088357	Early nodulin-like protein 8	Electron carrier activity

### QTL Analysis for Yield

**FIGURE 5:**
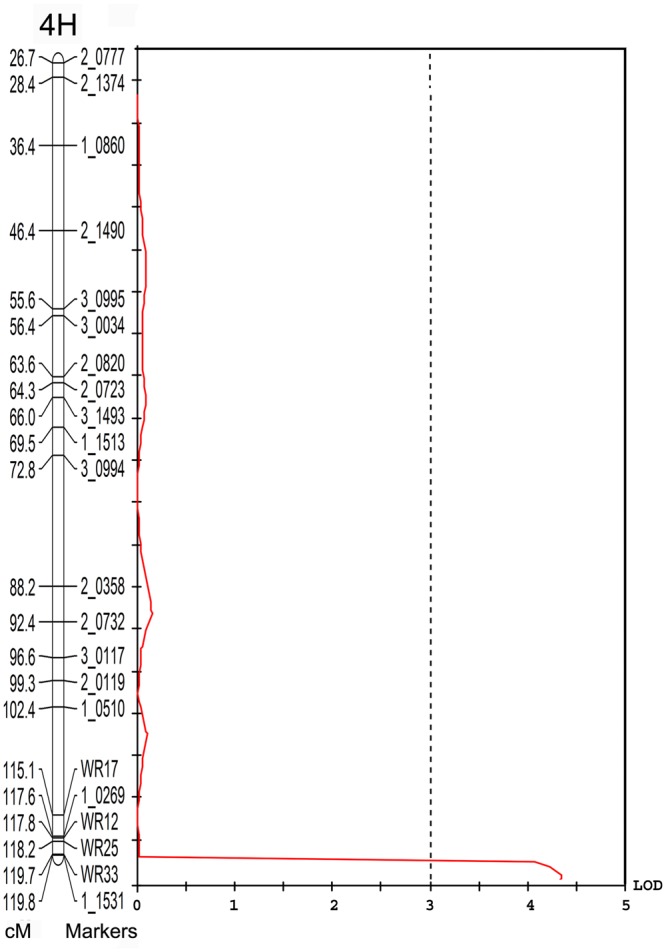
Quantitative trait loci associated with the 4H marker WR33 (LOD = 4) for grain yield in the field in the V-V-Y × Buloke DH population. The *X*-axis is the LOD score and the *Y*-axis is the markers and genetic distance (Kosambi cM) of chromosome 4H.

Individual yield data from the V-V-Y × Buloke DH population and the parental lines were collected. The average yield in the mutant V-V-Y and Vlamingh was 3755 ± 246.7 and 4194 ± 249.6 kg ha^-1^. The average yield of Buloke was 4340 ± 254 kg ha^-1^. The yield of the DH lines varied from 470 to 4953 kg ha^-1^. QTL analysis was performed to explore the genetic loci that control yield. A QTL located on chromosome 4H linked with marker WR33 explained 10.4% of the yield variation with LOD = 4 (**Figure 5**). This QTL is coincident with the *vvy* gene interval responsible for the thermo-inducible chlorophyll-deficient trait.

## Discussion

Various leaf color mutants have been described in barley (Gálová et al., 2000) but only a few genes controlling leaf color have been well characterized (Liu Z.L. et al., 2008; Qin et al., 2015; Shmakov et al., 2016). In this study, we showed that V-V-Y is a thermo-inducible chlorophyll-deficient mutant at the seeding and tillering stages. The genetic analysis showed that the thermo-inducible chlorophyll-deficient trait is controlled by a single mutant gene (*vvy*). Preliminary mapping showed that the *vvy* gene is located on the distal region of the long arm of chromosome 4H, which was further narrowed to a physical distance of 0.428 Mb by fine mapping, covering 11 annotated genes (Mascher et al., 2017). This is the first report of fine mapping a thermo-inducible chlorophyll-deficient gene in barley.

Several temperature-sensitive chlorophyll synthesis and regulation genes have been cloned. The *tcd5* (LOC_Os05g34040) gene, encoding a monooxygenase, is responsible for albino leaves at low temperatures in rice (Wang Y. et al., 2016). The *NOA1* gene (LOC_Os02g0104700) encodes a circularly permuted GTPase (cGTPase) that can regulate chlorophyll synthesis in a temperature-dependent manner (Yang et al., 2011). The *OsGluRS* gene on chromosome 2, encoding glutamyl-Trna synthetase, is responsible for high-temperature-induced yellow leaves in rice (Liu et al., 2007). Recently, the thermo-sensitive chlorophyll deficit 1 *(tscd1*) gene was mapped in a 34.95 kb region on the long arm of chromosome 2, which controls low-temperature-induced yellow leaves at the seeding and tillering stages in rice (Liu et al., 2015). In *Arabidopsis thaliana*, missense mutations in CHLOROPHYLL SYNTHASE (*chlg1-1*) (Lin et al., 2014) and CHLOROPHYLL DEPHYTYLASE1 (*cld1-1*) (Lin et al., 2016) were identified as responsible for the light-dependent, heat-induced cotyledon-bleaching phenotype. However, of the 11 candidate genes in this study, none was an orthologue of the cloned temperature-dependent chlorophyll biosynthesis and regulation gene.

The chlorophyll biosynthetic deficiency is one of the main reasons for low chlorophyll content in many plants (Zhou Y. et al., 2013). Yellow–green leaf is a type of mutant in rice; *ygl1* (Wu et al., 2007), *ygl2* (Chen et al., 2013), *ygl3* (Tian et al., 2013), *ygl6* (Shi et al., 2015), *ygl7* (allele of the *OsChlD* gene) (Deng et al., 2014), *ygl8* (Zhu et al., 2016), *ygl9* (Wang Z.-W. et al., 2016), and *ygl138* (Zhang et al., 2013) have been cloned, and most of these genes are involved in the chlorophyll biosynthesis process. However, none of the candidate genes of *vvy* are orthologous of the cloned rice yellow–green leaf genes.

The chlorophyll biosynthesis pathways have been well characterized in higher plants (Tanaka and Tanaka, 2007; Hörtensteiner and Kräutler, 2011). At least 27 genes covering 15 steps are involved in chlorophyll biosynthesis from glutamyl-Trna to chlorophyll *a* and chlorophyll *b* (Beale, 2005; Nagata et al., 2005). However, none of the candidate genes identified in this study was homologous to these 27 genes. Low chlorophyll contents might also result from deficient signal transduction between the nucleus and chloroplast (Zhou K. et al., 2013), restrained heme feedback (Terry and Kendrick, 1999), impaired synthesis and importation of chloroplast proteins (Gothandam et al., 2005; Miura et al., 2007) or harmful photooxidation (Richter et al., 2013). However, no known structural or transcription factor gene involved in the chlorophyll pathway was found in the target region. It is reasonable to speculate that a new gene for chlorophyll biosynthesis and regulation is involved in V-V-Y. Expression analysis of the annotated genes in V-V-Y and Vlamingh may provide more information for identification of the candidate gene, especially how these genes respond to high temperature treatment.

Heat stress during the reproductive and grain-filling phases is a severe threat to barley and wheat production. Chloroplasts, as the sensors to heat stress, are the major target of thermal damage in plants (Sun and Guo, 2016). Studies in barley (Havaux and Tardy, 1999), wheat (Mohammadi et al., 2009) and bentgrass (Jespersen et al., 2016; Rossi et al., 2017) indicated that chlorophyll losses with limited reductions in photosynthesis might be an adaptive response to high light and heat stress. The rice *tscd1* mutant can mature normally without any significant effects on key related-yield traits (Liu et al., 2015). In this study, the *vvy* gene interval overlapped with a QTL for barley yield under typical Mediterranean climate conditions with terminal drought and heat stresses. Heat stress can severely damage photosystem II (PSII), the most heat-sensitive photosynthetic apparatus within chloroplast thylakoid membrane protein complexes involved in photosynthetic electron transfer and ATP (Sun and Guo, 2016). Thus, we propose that the autonomic deficient chlorophyll biosynthesis under temperature stress might be a protective mechanism for crops to develop an adaptive response mechanism to temperature stress.

In most cases, leaf color mutants will retard plant growth (Wu et al., 2007; Jiang et al., 2012; Qin et al., 2015). However, some mutants can mature normally without any significant effect on yield-related traits (Chen et al., 2007; Liu et al., 2015). In the present study, the mutant V-V-Y leaves took longer to senesce than its wild parent under short period (2 days) of extreme high temperature (over 36°C) and thus maintained higher grain plumpness. The thermo-sensitive mutant may provide an ideal system for exploring why the reduction in chlorophyll content is an adaptive response to heat stress. It may also provide an opportunity to develop dynamic varieties that are temperature-sensitive to chlorophyll content with the potential to improve crop heat tolerance. However, the mutant gene could not provide yield advantage under a typical Mediterranean climate conditions with constant terminal drought and heat stresses. Thus, further research is required to understand the impact of the mutant gene on other yield components under both normal and heat stress conditions.

## Author Contributions

Project concept and design: CL, YX, WZ, and FY. Mutant identification: DW and CL. Population development: SB, SW, and X-QZ. Field trial: SB, SW, and X-QZ. Genotyping: RW, FY, X-QZ, CT, and CL. Wrote the MS: RW, YX, CL, and WZ.

## Conflict of Interest Statement

The authors declare that the research was conducted in the absence of any commercial or financial relationships that could be construed as a potential conflict of interest.

## Footnotes

1 http://www.uniprot.org/

2 http://www.ebi.ac.uk/interpro/

3 www.cbs.dtu.dk/services/TargetP
